# A Green Computing Business Aggregation Strategy for Low Earth Orbit Satellite Networks

**DOI:** 10.3390/s24248184

**Published:** 2024-12-21

**Authors:** Bo Wang, Jiaqi Lv, Dongyan Huang, Zelin Lu, Yuhang Fang

**Affiliations:** School of Information and Communication, Guilin University of Electronic Technology, 1 Xiamen Road, Guilin 541004, China; wangbo@guet.edu.cn (B.W.); ljq576453618@mails.guet.edu.cn (J.L.); lzl90648@outlook.com (Z.L.); 1972564665@mails.guet.edu.cn (Y.F.)

**Keywords:** LSNs, business aggregation, double DQN, energy efficiency, green computing

## Abstract

This paper proposes a green computing strategy for low Earth orbit (LEO) satellite networks (LSNs), addressing energy efficiency and delay optimization in dynamic and energy-constrained environments. By integrating a Markov Decision Process (MDP) with a Double Deep Q-Network (Double DQN) and introducing the Energy–Delay Ratio (EDR) metric, this study effectively quantifies and balances energy savings with delay costs. Simulations demonstrate significant energy savings, with reductions of up to 47.87% under low business volumes, accompanied by a minimal delay increase of only 0.0161 s. For medium business volumes, energy savings reach 26.75%, with a delay increase of 0.0189 s, while high business volumes achieve a 4.36% energy reduction and a delay increase of 0.0299 s. These results highlight the proposed strategy’s ability to effectively balance energy efficiency and delay, showcasing its adaptability and suitability for sustainable operations in LEO satellite networks under varying traffic loads.

## 1. Introduction

The 6th generation mobile networks (6G) are expected to offer higher data transmission rates, broader coverage, and reduced latency to meet the extensive future demands in information transmission and processing [1]. Low Earth Orbit (LEO) satellite networks (LSNs) provide full coverage for remote regions and, compared to other satellite systems, offer lower latencies, making them a critical component of 6G [2].

Despite these advantages, LSNs face significant challenges due to energy limitations, as LEO satellites rely on solar cells for power, leading to unstable energy supplies as satellites alternate between shadow and sunlight periods. This energy limitation affects the continuous operation and service reliability of LSNs [3].

To address these energy constraints, efficient solar energy acquisition is crucial. Perovskite-based solar cells have shown potential for improving energy conversion in space applications by increasing solar radiation absorption through optimized heat stimulation [4]. Additionally, effective onboard energy management can further optimize performance. For instance, managing energy usage across sunlit and shadow periods enhances transmission efficiency and coverage in high-mobility, large-scale connection scenarios [5].

Existing research shows that business aggregation can significantly reduce network energy consumption. The literature [6] points out that due to differences in business needs in different areas, about 45% of LEO satellite nodes can be shut down in areas with small business needs, thereby achieving energy saving effects. However, this study failed to consider how to deal with the resulting increase in latency when node shutdown is performed. Specifically, when some satellite nodes are shut down, services will be forwarded to other nodes, which may cause data transmission delays to increase and affect the overall performance of the system. Therefore, how to balance system delay while optimizing energy efficiency has become an important research issue.

Reference [7] further discusses the issue of saving network energy consumption through the establishment of dynamic inter-satellite links. This method avoids unnecessary link idleness by establishing link forwarding services on demand, thereby improving energy efficiency. However, ref. [7] failed to quantify the delay cost caused by such dynamic link establishment. Although this method effectively reduces energy consumption, the latency cost associated with frequent link establishment under high-load conditions has not been quantified, posing a significant challenge in balancing energy savings and delay performance.

To address the shortcomings identified in references [6,7], this paper combines their research insights and proposes a business aggregation and resource scheduling strategy based on reinforcement learning. Unlike these studies, this paper introduces a novel metric to quantify the trade-off between latency cost and energy savings, thereby addressing the lack of such quantification in the previous works. This strategy not only aims to reduce energy consumption while optimizing system delay but also takes into account green computing and real-time requirements. The work and contributions of this paper are as follows: First, an energy optimization strategy is proposed under the three-layer network architecture of the control satellite layer, business satellite layer, and user layer. An Energy–Delay Ratio (EDR) indicator is introduced to evaluate the effects of business aggregation optimization. Secondly, the Optimal Consolidation Strategy for Business Efficiency (OCSBE) is proposed. Finally, the effectiveness of aggregation strategies is verified through simulation analysis.

## 2. System Model

### 2.1. Network Model

**Figure 1:**
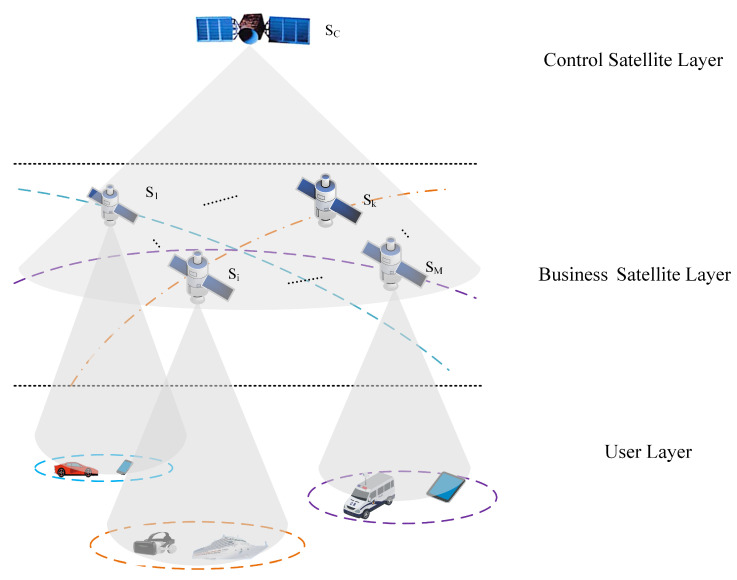
Layered architecture of LSNs showing control, business, and user layers.

The layered LSN network architecture is shown in Figure 1. The control satellite layer is composed of high-orbit satellites denoted as $S_{c}$, responsible for making aggregation decisions for the service satellites. The business satellite layer consists of *M* LEO satellites in low Earth orbit, responsible for handling specific businesses. We define the set of business satellites as $S=\{S_{1},\ldots ,S_{i},\ldots ,S_{k},\ldots ,S_{M}\}$, where $S_{i}$ and $S_{k}$ represent any arbitrary business satellites. The user layer consists of various types of terminals located on the ground, which generate businesses and transmit them to the business satellites covering their region.

The set of businesses received by the business satellite layer is defined as $B=\left\{B_{1},...,B_{i},...,B_{k},...,B_{M}\right\}$, where $B_{i}$ and $B_{k}$ are the businesses received by satellites $S_{i}$ and $S_{k}$, respectively. The set of business counts received by the business satellites is $N=\{N_{1},\ldots ,N_{i},\ldots ,N_{k},\ldots ,N_{M}\}$, where $N_{i}$ and $N_{k}$ represent the number of businesses received by satellites $S_{i}$ and $S_{k}$, respectively. We define the sequence of businesses received by $S_{i}$ as $B_{i}=\left\{B_{i1},...,B_{ij},...,B_{iN_{i}}\right\}$, where $B_{ij}$ is the *j*-th business received by $S_{i}$. The attribute list for business $B_{ij}$ is defined as $B_{ij}=\left\{B_{ij},\tau_{ij}^{\max}\right\}$, where $B_{ij}$ is the data size of business $B_{ij}$, in bits, and $\tau_{ij}^{\max}$ is its maximum tolerable delay, in seconds.

The LEO satellite orbit model is given as follows:

(1) r(t)=(R+h)[1−ecosα(t)]

where *R* is the Earth’s radius, *h* is the altitude of the satellite, *e* is the orbital eccentricity, and α(t) is the true anomaly angle.

The distance between two satellites $S_{i}$ and $S_{k}$ is calculated as follows:

(2) $$D_{ik}=\sqrt{d_{ik}^{2}+{\left(h_{i}-h_{k}\right)}^{2}}$$

where $d_{ik}$ is the spherical distance between $S_{i}$ and $S_{k}$, and $h_{i}$, $h_{k}$ are their respective orbital heights.

### 2.2. Communication Model

The uplink channel capacity between a ground user and $S_{i}$ is:

(3) $$C_{GS_{i}}=B_{GS_{i}}\log_{2}\left(1+\frac{P_{tx}G_{tx}H_{GS_{i}}}{N_{GS_{i}}}\right)$$

where $B_{GS_{i}}$ is the channel bandwidth, $P_{tx}$ and $G_{tx}$ are the transmit power and antenna gain of the user equipment, $H_{GS_{i}}$ is the channel gain, and $N_{GS_{i}}$ is the noise power [8].

The transmission delay for business $B_{ij}$ from the user to $S_{i}$ is given as follows:

(4) $$\tau_{ij}=\frac{B_{ij}}{C_{GS_{i}}}$$

The channel capacity between $S_{i}$ and $S_{k}$ is:

(5) $$C_{S_{i}S_{k}}=B_{S_{i}S_{k}}\log_{2}\left(1+\frac{P_{TX}G_{TX}H_{S_{i}S_{k}}}{N_{S_{i}S_{k}}}\right)$$

where $B_{S_{i}S_{k}}$ is the bandwidth, $P_{TX}$ is the transmission power, $G_{TX}$ is the antenna gain of $S_{i}$, $H_{S_{i}S_{k}}$ is the channel gain between $S_{i}$ and $S_{k}$, and $N_{S_{i}S_{k}}$ is the noise power.

The delay required for transmitting $B_{ij}$ from $S_{i}$ to $S_{k}$ is as follows:

(6) $$\tau_{ijk}=\frac{B_{ij}}{C_{S_{i}S_{k}}}$$

Since the computational results to be sent back to the ground are relatively small, the energy consumption and delay for the return transmission are negligible.

### 2.3. Computation Model

For a business $B_{ij}$ being processed at $S_{i}$, the computation delay, assuming the existence of a ta business queue, is:

(7) $$\tau_{ij}^{c}=\sum_{l=1}^{j-1}\frac{B_{il}\cdot \kappa }{f}+\frac{B_{ij}\cdot \kappa }{f}$$

where *f* is the CPU processor frequency of $S_{i}$ (in cycles/s), and κ represents the computation capability (in cycles/bit).

The total delay for $S_{i}$ to process all businesses $B_{i}$ is as follows:

(8) $$\tau_{i}=\sum_{j=1}^{N_{i}}\frac{B_{ij}\cdot \kappa }{f}$$

The energy consumed for computation at $S_{i}$ is:

(9) $$E_{c}^{i}=P_{c}\cdot \tau_{i}$$

where $P_{c}$ is the computation power of $S_{i}$.

## 3. Problem Modeling

Based on the operational modes of satellites, the working state of $S_{i}$ can be divided into three states: active, idle, and sleep. In its active state, the satellite performs business transmission and computation functions, consuming the highest power, with average power consumption for transmission, reception, and computation modules denoted as $P_{T}^{a}$, $P_{R}^{a}$, and $P_{C}^{a}$, respectively. In the idle state, the satellite maintains a low-power mode for communication and computation modules, with power levels $P_{T}^{idle}$, $P_{R}^{idle}$, and $P_{C}^{idle}$. In the sleep state, the computation module is turned off to save power, with $P_{C}^{sleep}=0$, though communication functions remain active for rapid response [9,10].

In specific scenarios, such as low business volume at night or in sparsely populated regions, certain satellites are underutilized. To enhance energy efficiency, a business aggregation strategy can be employed to consolidate businesses on a subset of satellites, allowing others to enter a sleep state.

**Figure 2:**
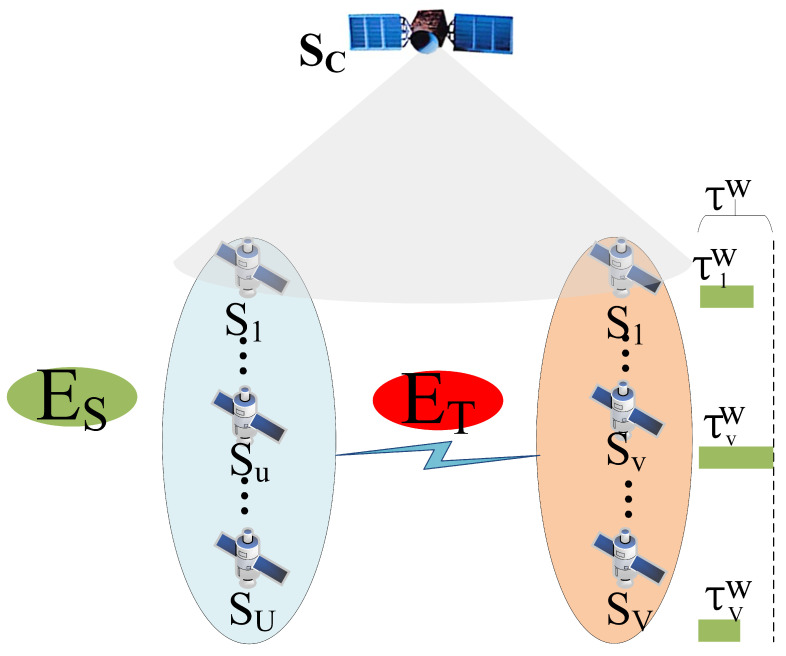
Business satellite load aggregation diagram.

As illustrated in Figure 2, the LSNs concentrate business processing on a subset of service satellites, allowing other satellites to enter a sleep state. We define the set of sleeping satellites as $S^{S}=\left\{S_{1},\ldots ,S_{u},\ldots ,S_{U}\right\}$ and working satellites as $S^{W}=\left\{S_{1},\ldots ,S_{v},\ldots ,S_{V}\right\}$, where $S_{u}$ and $S_{v}$ are any sleep satellites and working satellites, respectively, and *U* and *V* represent the number of sleeping and working satellites, respectively, such that U+V=M.

The energy consumption for transferring a business $B_{uj}$ from a sleeping satellite $S_{u}$ to a working satellite $S_{v}$ is:

(10) $$E_{T}^{ujv}=\left(P_{T}^{a}+P_{R}^{a}\right)\cdot \tau_{ujv}$$

where $B_{uj}$ denotes the *j*-th business of the *u*-th sleeping satellite, and $\tau_{ujv}$ is the transmission duration of $B_{uj}$ from the *u*-th sleeping satellite to the *v*-th working satellite.

The total communication energy for aggregating businesses from $S^{S}$ to $S^{W}$ is:

(11) $$E_{T}=\sum_{u=1}^{U}\sum_{j=1}^{N_{u}}E_{T}^{ujv}$$

where $N_{u}$ is the number of businesses of the *u*-th sleep satellite.

The energy savings for a sleeping satellite $S_{u}$ are:

(12) $$E_{s}^{u}=P_{C}^{idle}\cdot \tau^{\prime }$$

where $\tau^{\prime }$ is the duration of the sleep period.

The total energy savings for all sleeping satellites $S^{S}$ are:

(13) $$E_{s}=\sum_{u=1}^{U}E_{s}^{u}$$

The net energy saved by business aggregation is:

(14) $$\Delta E=E_{s}-E_{T}$$

After receiving businesses from sleeping satellites, the working satellite $S_{v}$ updates its business queue. Suppose business $B_{uj}$ is assigned a sequence number $N_{a}$ upon insertion; then the updated queue for $S_{v}$ is $B_{v}^{\prime }=\left\{B_{v1},\ldots ,B_{vN_{v}},\ldots ,B_{vN_{a}},\ldots ,B_{vN_{v}^{\prime }}\right\}$, where $N_{v}^{\prime }$ represents the total number of businesses in $S_{v}$ after aggregation.

The computation delay for $S_{v}$ processing $B_{vN_{a}}$ after aggregation is as follows:

(15) $$\tau_{ujv}^{c}=\sum_{l=1}^{N_{a}}\frac{B_{vl}\cdot \kappa }{f}$$

The total computation delay for $S_{v}$ after aggregation is as follows:

(16) $$\tau_{v}^{w}=\sum_{m=1}^{N_{v}^{\prime }}\frac{B_{vm}\cdot \kappa }{f}$$

The maximum delay for all working satellites after aggregation is as follows:

(17) $$\tau^{w}=\max\left(\tau_{1}^{w},\ldots ,\tau_{v}^{w},\ldots ,\tau_{V}^{w}\right)$$

The maximum delay without aggregation is as follows:

(18) $$\tau^{o}=\max\left(\tau_{1},\ldots ,\tau_{i},\ldots ,\tau_{M}\right)$$

The delay increment due to aggregation is as follows:

(19) $$\Delta D=\tau^{w}-\tau^{o}$$

In order to comprehensively evaluate the impact of energy savings and delay increase, this paper defines Energy–Delay Ratio (EDR) as follows:

(20) $$EDR=\frac{\Delta E}{\Delta D}$$

where EDR represents the energy savings per unit delay increase (in J/s).

The optimization objective of the business aggregation strategy is defined as follows:

(21) $$\begin{array}{c} \mathrm{maximize}\;\mathrm{EDR}\\ \mathrm{subject}\;\mathrm{to}:\\ \tau_{uj}+\tau_{ujv}+\tau_{ujv}^{c}<\tau_{uj}^{\max}\;\left(a\right)\\ \sum_{v=1}^{M}x_{ujv}=1\;\left(b\right)\\ 1\leq U\leq M\;\left(c\right)\\ U+V=M\;\left(d\right) \end{array}$$

where constraint (a) ensures that the business processing time is less than the maximum tolerable delay for the business; constraint (b) ensures that each business $B_{uj}$ is assigned only once, with $x_{ujv}$ taking a value of 0 or 1 to indicate whether the business is assigned to business satellite $S_{v}$; constraint (c) defines the range of the number of working satellites; and constraint (d) ensures that the sum of working and sleeping satellites equals the total number of business satellites.

## 4. Problem Solution

Optimization involves both continuous and discrete variables, which is a mixed-integer programming problem. The constraints are nonlinear, so this problem can be classified as a mixed integer nonlinear programming problem. For the above type of complex optimization problems, this paper divides them into two sub-problems:

- Sub-problem 1: Determining the set of sleeping satellites
- Sub-problem 2: aggregating businesses from sleeping satellites to working satellites

**Figure 3:**
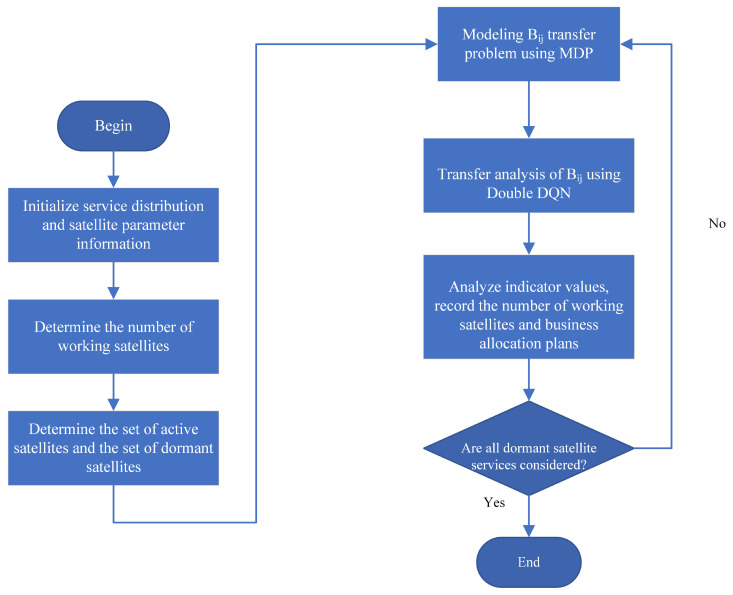
Business aggregation flow chart.

This paper proposes an optimal integration strategy for optimizing business efficiency (OCSBE), which aims to aggregate business onto service satellites. The specific process is shown in Figure 3.

- Step 1: Initialization. The control satellite initializes the process by collecting information on service distribution and satellite parameters.
- Step 2: Determine the number of working satellites. Based on the current service volume, determine the number of satellites that will remain active.
- Step 3: Determine the set of active satellites and the set of dormant satellites. Classify satellites into active and dormant sets based on service demand.
- Step 4: Model the $B_{uj}$ transfer problem using MDP. Use a Markov Decision Process (MDP) to model the transfer problem for $B_{uj}$.
- Step 5: Perform transfer analysis of $B_{uj}$ using double DQN. Conduct transfer analysis for $B_{uj}$ using a Double Deep Q-Network (Double DQN).
- Step 6: Analyze indicator values, record the number of working satellites and business aggregation plans. Analyze indicator values, and record the number of active satellites and the business aggregation scheme.
- Step 7: Check if all dormant satellite services are considered. Verify if all services for dormant satellites have been considered. If not, return to continue the process; if yes, end the process.

### 4.1. Determining the Set of Sleeping Satellites

For Sub-problem 1, this paper adopts the Capacity-Driven Sleep Satellite Quantity (CDSSQ) mechanism to determine the number of sleeping satellites. The design is as follows:

Sorting the satellites: First, sort the business satellites in ascending order based on their business volume to obtain the list $S^{\prime }=\left\{S_{1},S_{2},\ldots ,S_{v},\ldots ,S_{M}\right\}$.
Calculating the number of working satellites: Determine the number of working satellites *V* using the relationship between the current business volume $B^{c}$ and the system’s maximum capacity $B^{\max}$ as per Equation (22):
  (22) $$V=\left\lceil \frac{B^{c}}{B^{\max}}\cdot M\right\rceil$$
  where $B^{c}$ represents the current total business volume of $S^{\prime }$; $B^{\max}$ is the maximum computable business volume per satellite (measured in bits); and ⌈·⌉ denotes the ceiling function.
Determining satellite sets: Based on the calculated number *V* and the sorted list $S^{\prime }$, identify the set of working satellites $S^{W}$ and the set of sleeping satellites $S^{S}$.

### 4.2. Aggregating Businesses

We address this sub-problem by modeling it as a Markov Decision Process (MDP) and employing a Double Deep Q-Network (Double DQN) for business reassignment.

#### 4.2.1. MDP Element Group

- We define the elements of the MDP as follows:
  1. State space ($s_{t}$): $s_{t}=\left\{V,B_{1},\ldots ,B_{u},\ldots ,B_{v},\ldots ,B_{M}\right\}$, where $B_{i}$ represents the current business volume of satellite $S_{i}$.
  2. Action space ($a_{t}$): $a_{t}=\left\{a_{1},\ldots ,a_{v},\ldots ,a_{V}\right\}$. Each action $a_{k}$ denotes assigning business $B_{uj}$ to satellite $S_{v}$.
  3. Transition probability ($P(s_{t+1}|s_{t},a_{t})$): The probability of assigning business $B_{uj}$ to satellite $S_{v}$ is represented as follows: $P(B_{v}+B_{uj}|B_{v},a_{v})$. This denotes the probability of satellite $S_{v}$ transitioning from state $B_{v}$ to state $B_{v}+B_{uj}$ after action $a_{v}$.
  4. Reward function ($R(s_{t},a_{t})$): The reward for assigning business $B_{uj}$ to satellite $S_{v}$ is defined as $R(B_{v},a_{v})$.
  5. Discount factor (γ): γ is a discount factor between 0 and 1 that balances the importance of current and future rewards.

#### 4.2.2. Double DQN

The double DQN approach is designed to reduce the overestimation of Q-values by decoupling the action selection from the action evaluation. The implementation details are as follows:

Utilize Double DQN to learn the optimal business aggregation policy that maximizes cumulative rewards. Double DQN employs two Q-networks to mitigate the overestimation of Q-values:

- Online network ($Q_{o}\left(s_{t},a_{t};\theta \right)$): The primary network used for selecting actions.
- Target network ($Q_{T}\left(s_{t},a_{t};\theta^{-}\right)$): A secondary network with parameters $\theta^{-}$ that are periodically updated to stabilize training.
- Action selection using ε-greedy strategy: Based on the ε-greedy policy, an action $a_{v}$ is selected in a given state $s_{t}$ as per the following:
  (23) $$a_{v}=\left\{\begin{array}{cc} \mathrm{random}\;\mathrm{action} & \mathrm{if}\;\mathrm{random}<\varepsilon \\ \arg\max_{a}Q_{o}\left(s_{t},a;\theta \right) & \mathrm{otherwise} \end{array}\right.$$
  where ε is a probability between 0 and 1 representing the likelihood of choosing a random action for exploration, and $Q_{o}\left(s_{t},a;\theta \right)$ is the action–value function of the online network.
- Calculating transition probabilities: The probability of transferring business $B_{uj}$ to satellite $S_{v}$ is computed as:
  (24) $$P\left(B_{v}+B_{uj}|B_{v},a_{v}\right)=\left\{\begin{array}{cc} \frac{B_{v}^{\max}-B_{uj}-B_{v}}{B_{v}^{\max}} & \mathrm{if}\;B_{uj}+B_{v}<B_{v}^{\max}\\ 0 & \mathrm{otherwise} \end{array}\right.$$
  where $B_{v}^{\max}$ is the maximum business volume that satellite $S_{v}$ can handle, and $B_{v}$ represents the current business volume of satellite $S_{v}$.
- Reward calculation: The reward for taking action $a_{v}$ in state $B_{v}$ is calculated as:
  (25) $$R\left(B_{v},a_{v}\right)=\frac{\tau_{ujv}\cdot P_{C}^{\mathrm{idle}}-\tau_{ujv}\cdot \left(P_{T}^{a}+P_{R}^{a}\right)}{\tau_{ujv}+\tau_{ujv}^{c}-\tau_{uj}^{c}}$$
  where $\tau_{ujv}$ is the energy difference associated with the business transfer; $P_{C}^{idle}$ is the idle power consumption; $P_{T}^{a}$ and $P_{R}^{a}$ are the transmission and reception power consumptions, respectively; and $\tau_{ujv}^{c}$ and $\tau_{uj}^{c}$ represent delay components.
- Updating the Q-values using the Bellman equation: We update the Q-value of the online network using the Bellman equation as shown:
  (26) $$Q_{o}\left(s_{t},a_{v};\theta \right)\leftarrow Q_{p}\left(s_{t},a_{v};\theta \right)+\alpha \left[R\left(B_{v},a_{v}\right)+\gamma \max_{a^{\prime }}Q_{T}\left(s_{t+1},a^{\prime };\theta^{-}\right)-Q_{p}\left(s_{t},a_{v};\theta \right)\right]$$
  where $Q_{p}\left(s_{t},a_{v};\theta \right)$ is the previous Q-value before the update, α is the learning rate, γ is the discount factor, and $\theta^{-}$ are the parameters of the target network.
- Convergence check: Perform a convergence check to determine if the Q-values have stabilized:
  (27) $$\Delta Q=\left|Q_{o}\left(S_{t},a_{v};\theta \right)-Q_{p}\left(S_{t},a_{v};\theta \right)\right|<\delta$$
  where δ is a small threshold value. If ΔQ remains below δ for multiple consecutive updates, the Q-values are considered to have converged, indicating that the optimal business aggregation strategy for $B_{uj}$ has been found.

Repeat the above steps to make aggregation decisions for all businesses that need to be transferred, and form the final business-working satellite mapping.

## 5. Simulation Analysis

### 5.1. Simulation Parameter Design

Using the Systems Tool Kit (STK), a three-layer network architecture is modeled, with control satellites at 2000 km altitude and 18 orbital planes of business satellites (40 per plane) distributed between 500 and 1500 km in altitude. The power range of the entire functional module of the business satellite for sending intersatellite signals is 10 W–50 W [10], the power range of the entire functional module for receiving signals is 5 W–25 W [10], and the power range of the entire functional module for computing functions is 60 W–415 W [11]. The clock frequency of the satellite CPU is $2\times 10^{10}$ cycle/s [12]. The number of businesses ranges from 1000 to 9000, and these businesses are randomly distributed across different satellites [13]. The parameters of LSNs are shown in Table 1.

The Double DQN strategy parameters are listed in Table 2. The learning rate was set to 0.001, ensuring a stable parameter update step size, with an epsilon decay rate of 0.995 to balance exploration and exploitation phases. The discount factor γ was set to 0.99 to emphasize the importance of long-term rewards, and the training involved 500 episodes.

### 5.2. Simulation Scenario Analysis with STK

**Figure 4:**
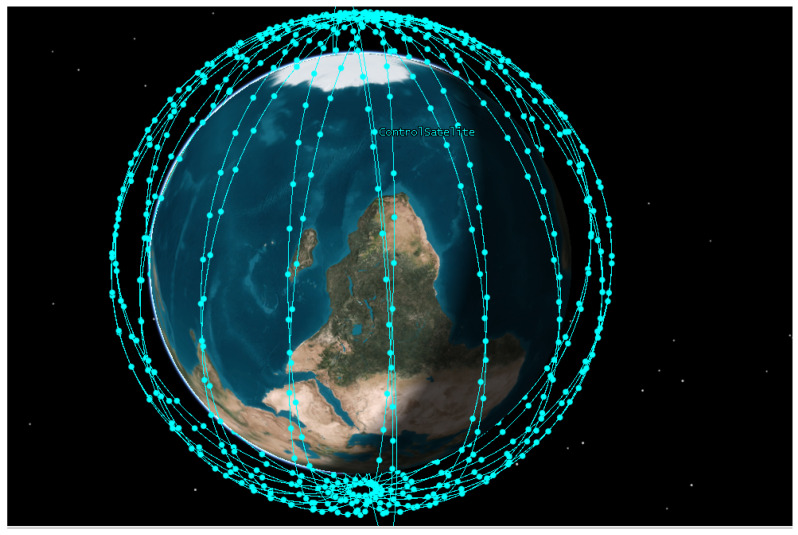
Satellite simulation scene map.

The simulation scenario constructed using STK is shown in Figure 4. This scenario is based on 18 orbital planes, with each orbital plane containing 40 satellites, forming a dense coverage network. This design ensures that, regardless of the satellite positions, connectivity can be provided to different areas of the Earth’s surface.

The control satellites operate at an altitude of 2000 km, with an orbital period of approximately two hours. Based on the analysis conducted using the STK simulation, the satellite network topology over a two-hour period was studied. First, the access duration of all satellites was analyzed, revealing an average access duration of approximately 10 min. Therefore, the two-hour period was divided into 12 intervals of 10 min each.

Based on the statistical average, the number of satellites achieving stable network configurations in each time interval was determined to be five. Therefore, time intervals with a stable network topology involving five satellites were selected for analysis. This number represents the average count of satellites with stable network topologies, calculated across multiple time intervals within a single operational cycle. However, as satellite networks evolve and more satellites are deployed, this number will naturally increase, allowing for a more comprehensive representation of real-world Low Earth Orbit Satellite Networks (LSNs).

### 5.3. Strategy Simulation and Analysis

**Figure 5:**
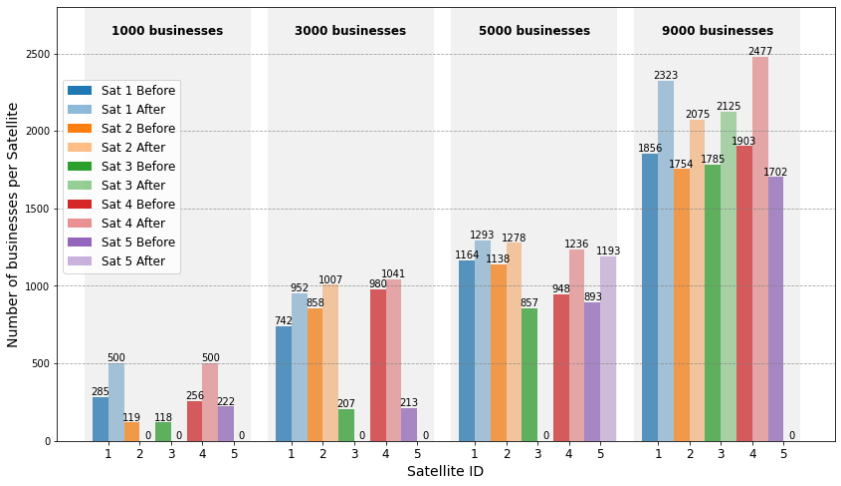
Performance of the recommended aggregation strategy.

Figure 5 illustrates the performance of the proposed strategy in business aggregation. Firstly, as the business volume increases, the number of satellites involved in processing also rises correspondingly to meet the growing demand, ensuring sufficient computational capacity. Secondly, the strategy tends to select satellites with initially higher business volumes as aggregation points. This approach effectively minimizes the transmission energy required during the aggregation process by leveraging satellites that are already heavily loaded. Lastly, after the business aggregation is complete, the load disparity among the working satellites is significantly reduced. This improvement can be attributed to the reward–punishment mechanism embedded in the strategy, which ensures a fair and efficient distribution of tasks. By balancing the workload, this mechanism not only maximizes the overall system efficiency but also enhances the stability and reliability of the network under varying traffic conditions.

To validate the performance advantage of the proposed strategy, this paper compares it with the following baseline strategies:

- Ant Colony Strategy (*AC*): This strategy generates aggregation decisions and resource aggregations by simulating ant colony behavior for global optimization.
- MDP-QL Strategy (*MDP-QL*): This strategy constructs a Q-table to generate aggregation decisions and resource aggregations, aiming to achieve global optimization.

**Figure 6:**
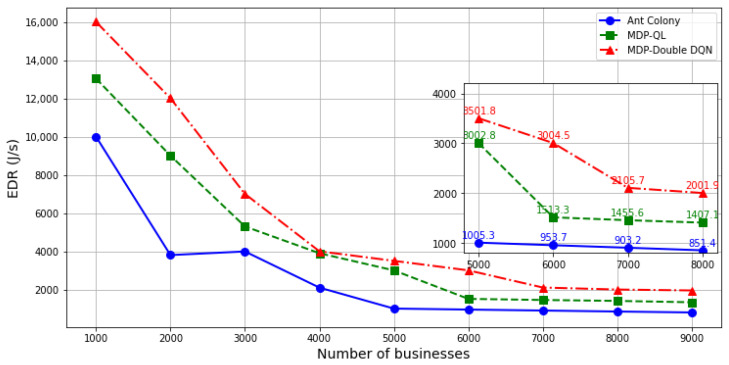
Energy–Delay Ratio (EDR) versus number of businesses for different optimization strategies.

Figure 6 analyzes the performance of different optimization strategies. Firstly, as the business volume increases, the overall optimization objectives of all strategies exhibit a downward trend and eventually converge. This decline is primarily caused by the increased transmission energy consumption and processing delay associated with higher business volumes, which leads to a reduction in the EDR value. Secondly, the proposed strategy consistently outperforms other strategies under varying business volume conditions. This advantage stems from the use of a reinforcement learning approach, which effectively balances the exploration and exploitation of optimal solutions in continuous state spaces, thereby achieving superior optimization results.

Table 3 shows that the proposed aggregation strategy performs best in low-traffic scenarios. When the business volume is low (e.g., 1000), the strategy achieves significant energy savings of 47.87% with a minimal delay increment of 0.0161 s. This highlights its efficiency in conserving energy while maintaining a low latency.

As the business volume increases, energy savings decrease significantly due to a reduction in the number of dormant satellites. For instance, at 5000 businesses, the energy reduction ratio drops to 11.34%, and at 9000, it further decreases to 4.36%. Simultaneously, the delay increment stabilizes, peaking at 0.0304 s at 7000 businesses and slightly falling to 0.0299 s at 9000, which can be attributed to the activation of more satellites to handle the traffic load. These results demonstrate the strategy’s effectiveness in low-traffic scenarios but also its limitations in high-traffic conditions.

## 6. Conclusions

This paper proposes a business aggregation strategy for Low Earth Orbit (LEO) Satellite Networks (LSNs) to address energy constraints by incorporating green computing principles. The strategy dynamically adjusts satellite operations based on business volume, enabling some satellites to enter a sleep state to conserve energy. A three-layer architecture, including control satellites, service satellites, and user devices, is utilized, with optimization achieved through a Markov Decision Process (MDP) and a Double Deep Q-Network (Double DQN).

The simulation results demonstrate a 47.87% energy reduction under low business volume and a 4.36% reduction under high business volume, showcasing its effectiveness, especially in low-traffic scenarios.

This research provides practical insights into energy-efficient management for LEO satellite networks and related energy-sensitive fields, such as data centers and smart grids. The proposed strategy reduces energy consumption, enhances resource efficiency, and supports the broader adoption of green computing and sustainable development globally. Moreover, one of the key visions of 6G is ubiquitous connectivity, which is intrinsically linked to the advancement of LEO satellite networks. As these networks evolve, energy optimization will become an inevitable consideration. Therefore, the strategy proposed in this paper offers valuable reference and guidance for the future development of LEO satellite networks, supporting their role in achieving the goals of 6G.

## Figures and Tables

**Table 1:** LSN parameters.

Parameter	Value
Control satellite altitude	2000 km
Business satellite altitude	500–1500 km
Number of control satellites	120
Number of business satellites	720
Number of businesses	1000–9000
Elevation angle	15.2–51.9
$P_{T}^{a}$	10–50 W
$P_{R}^{a}$	5–25 W
$P_{c}^{a}$	60–415 W
$P_{TX}^{idle}$	5–8 W
$P_{RX}^{idle}$	3–6 W
$P_{C}^{idle}$	45–55 W
$P_{tx}$	3–5 W
$P_{rx}$	1–3 W
$B_{GS_{i}}$	20–40 MHz
$B_{S_{i}S_{k}}$	20–40 MHz
$C_{S_{i}S_{k}}$	$2\times 10^{8}$ bit/s
*f*	$2\times 10^{10}$ cycle/s
$N_{GS_{i}}$	−140.4 dBm
$N_{S_{i}S_{k}}$	−110 dBm
κ	300 cycle/bit
$\tau^{\prime }$	1 s
$\tau_{ij}^{\max}$	0.6 s
$B_{ij}$	$1\times 10^{3}$ bit–$1\times 10^{6}$ bit

**Table 2:** Double DQN Strategy Parameters.

Parameter	Value
Learning rate (lr)	0.001
Epsilon decay	0.995
Discount factor (γ)	0.99
Episodes	500

**Table 3:** Delay increments and energy reduction ratios for different business volumes.

Number of Businesses	Delay Increment (s)	Energy Reduction Ratio
1000	0.0161	47.87%
3000	0.0227	22.01%
5000	0.0203	11.34%
7000	0.0304	7.83%
9000	0.0299	4.36%

## Data Availability

The data presented in this study are available on request from the corresponding author.
